# Melanoma-Associated Cancer-Testis Antigen 16 (CT16) Regulates the Expression of Apoptotic and Antiapoptotic Genes and Promotes Cell Survival

**DOI:** 10.1371/journal.pone.0045382

**Published:** 2012-09-21

**Authors:** Camilla Nylund, Pekka Rappu, Eveliina Pakula, Aleksi Heino, Laura Laato, Laura L. Elo, Pia Vihinen, Seppo Pyrhönen, Gethin R. Owen, Hannu Larjava, Markku Kallajoki, Jyrki Heino

**Affiliations:** 1 Department of Biochemistry and Food Chemistry, University of Turku, Turku, Finland; 2 Department of Mathematics, University of Turku, Turku, Finland; 3 Department of Oncology and Radiotherapy, Turku University Hospital, Turku, Finland; 4 Department of Oral, Biological and Medical Sciences, University of British Columbia, Vancouver, British Columbia, Canada; 5 Department of Pathology, University of Turku, Turku, Finland; Duke University Medical Center, United States of America

## Abstract

Cancer-testis (CT) antigens are predominantly expressed in testis or placenta, but absent in most adult tissues. During malignant transformation CT genes are often activated. CT antigen 16 (CT16, PAGE5) is frequently expressed in advanced melanoma but its biological function has been unknown. To examine the role of CT16 in cell survival we knocked it down in A2058 melanoma cells using specific siRNAs and exposed the cells to cancer drug cisplatin known to induce apoptosis. As a result, cell survival was markedly decreased. To study the effects of CT16 on cell survival in more detail, the cellular gene expression profiles were investigated after CT16 silencing in CT16 positive A2058 melanoma cells, as well as after CT16 overexpression in CT16 negative WM-266-4 melanoma cells. Among the 11 genes both upregulated by CT16 silencing and downregulated by CT16 overexpression or vice versa, 4 genes were potentially apoptotic or antiapoptotic genes. CT16 was recognized as a positive regulator of antiapoptotic metallothionein 2A and interleukin 8 genes, whereas it inhibited the expression of apoptosis inducing dickkopf 1 (DKK1) gene. In addition CT16 enhanced the expression of fatty acid binding protein 7, a known promoter of melanoma progression. The effect of CT16 on DKK1 expression was p53 independent. Furthermore, CT16 did not regulate apoptotic genes via DNA methylation. In twenty melanoma metastasis tissue samples average DKK1 mRNA level was shown to be significantly (p<0.05) lower in high CT16 expressing tumors (n = 3) when compared to the tumors with low CT16 expression (n = 17). Thus, our results indicate that CT16 promotes the survival of melanoma cells and is therefore a potential target for future drug development.

## Introduction

Cancer-testis antigens (CTAs) form a heterologous group of proteins that are expressed in gametes and trophoblasts [1]. CTdatabase of Ludwig Institute for Cancer Research (http://www.cta.lncc.br/index.php) [2] lists more than 140 CTAs. The CTAs can be divided to X-chromosome-encoded (X-CTAs) and non-X-chromosome-encoded CTAs (non-X-CTAs). The most common mechanism of CTA gene activation is promoter demethylation [1]. Gametogenesis and tumorigenesis have many corresponding and shared events, including global hypomethylation and CTA expression [3]. However, it is unclear, whether the expression of CTAs is merely a consequence of demethylation events common in tumorigenesis or whether the CTAs have an active role in promoting cancer progression.

According to the CTdatabase, many non-X-CTAs seem to have a role in spermatogenesis and sperm function in normal testis. In contrast, the biological role of X-CTAs is mostly unknown. The function of gene products encoded by two X-CTA families, MAGE and SSX, has been previously studied. MAGE-A antigens have been reported to directly interact with p53 and repress its function [4], [5]. Moreover, MAGE proteins have been shown to form a complex with a scaffolding protein, KRAB-associated protein 1 (KAP1) and to suppress p53-dependent apoptosis [6]. MAGE has also been demonstrated to interact with RING E3 ubiquitin ligases and to enhance the ubiquitination activity of RING domain proteins towards their targets, such as p53 [7]. The SSX family members that can fuse with SS18, as a result of a specific chromosomal translocation in human synovial sarcoma [8], have been suggested to act as transcriptional regulators [9]. Yeast two hybrid-screening and glutathione-S-transferase pull-down assays have shown that SSX2 protein binds to RAB3IP and SSX2IP proteins [10]. On the other hand, SSX proteins have been reported to colocalize with the members of polycomb group complex [11], and in later studies evidence of the role of SSX proteins in histone modification and DNA methylation have been found [12], [13].

CT16 (also known as PAGE5, GAGEE1, CT16.1) is an X-CTA and its closest relatives belong to the prostate-associated antigen family (PAGE genes), which in turn is related to XAGE and GAGE gene families [14]. CT16 was first reported in a study where the Unigene database was searched for gene clusters containing expressed sequence tags originated solely from both normal human testis and tumor cDNA libraries. The expression of CT16 in melanomas as well as in lung and renal cancers was shown at the mRNA level [15]. More recently we reported the expression of CT16 mRNA in 11 out of 22 melanoma skin metastasis. Moreover, we showed that despite the homology to known prostate-associated antigens CT16 is not expressed in primary prostate cancer [16]. We also developed a sensitive assay to measure CT16 directly from patient sera and showed that 14 out of 23 (61%) patients with metastatic melanoma had detectable CT16 levels [16].

The biological role of CT16 has been unknown, but there are some reports about the putative functions of the CT16 homologs. One GAGE family member having 30% identity with CT16 has been reported to inhibit apoptosis and to interact with nucleophosmin/B23 and interferon regulatory factor 1 [17]. Similarly, it has recently been shown that prostate cancer associated antigen 4 (PAGE4), which has 43% identity to CT16, is an anti-apoptotic protein [18]. In the same study, the authors also reported that PAGE4 is an intrinsically disordered protein (IDP) that binds to GC-rich sequences in double-stranded DNA [18]. The NMR analysis of CT16 has also indicated that it is an IDP [19]. In fact, the sequence analysis with IUPred algorithm (http://iupred.enzim.hu) [20] predicts that all proteins related to CT16 are intrinsically disordered. IDPs are proteins that do not form a rigid 3-D structure in physiological conditions [21]. Moreover, a recent *in silico* analysis suggests that most CTAs are IDPs [22]. Approximately 25% of the mammalian proteins are predicted to be IDPs, and about 75% of the mammalian signaling proteins are predicted to have long disordered regions [23]. It has been suggested that IDPs often have multiple functions, a property termed as moonlighting [24]. Thus, also CT16 and the CT16-related CTAs may have different functions depending on the biological context and cellular localization.

In this paper we have studied the role of CT16 in melanoma. We show that silencing of CT16 with siRNAs enhances cisplatin induced cell death. Transcriptome analysis utilizing CT16 overexpression and knockdown revealed that CT16 negatively regulates, probably indirectly, expression of an antiapoptotic protein DKK1. We demonstrate that this regulation is p53 independent and CT16 has no effect on the methylation of the survival regulating genes. We also suggest that human melanoma skin metastasis samples with high CT16 mRNA level may have less DKK1 mRNA than those with low CT16 mRNA level.

## Results

### CT16 is expressed in melanoma metastasis and melanoma cell lines at protein level

**Figure 1:**
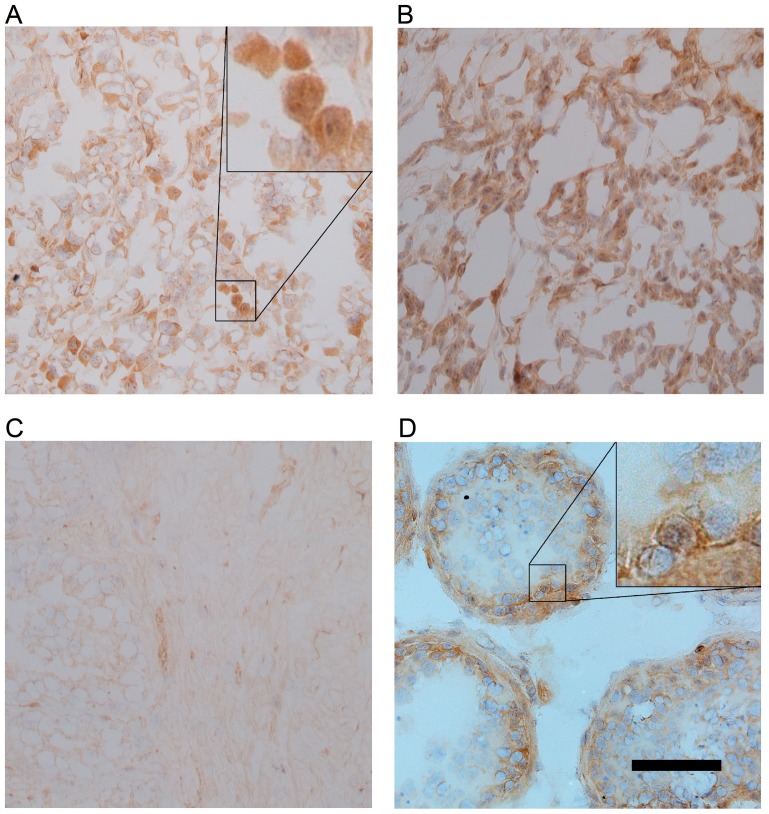
CT16 location in frozen tissue samples. CT16 was detected in the tissue samples with CT16-specific antibody visualized by Vectastain ABC kit and diaminobenzidine, and the sections were counterstained with hematoxylin. (A, B) Melanoma skin metastasis samples from two patients. (C) Primary prostate cancer (negative control). (D) Testis sample (positive control). Both cytoplasmic and nuclear CT16-specific staining is visible in the magnified area (inset) of the melanoma skin metastasis sample. In the testis sample the magnification shows CT16-specific staining of spermatogonia. The scale bar in the testis image represents the magnification as 150 µm for all images.

We have previously reported the production of specific polyclonal antibody against human recombinant CT16 [16]. Here, the antibody was used to verify CT16 expression at protein level in melanoma skin metastasis. The frozen tissue samples were studied using immunohistochemistry. CT16 expression was only visible in melanoma metastasis tissues that expressed CT16 mRNA (Figure 1A, B). In addition, the amount of CT16-specific staining correlated well with the mRNA levels (Figure S1). The primary prostate cancer samples used as negative controls did not show any staining for CT16 (Figure 1C). A testis sample was used as a positive control and CT16 was located in the basal compartment of spermatogenic epithelium, mainly in spermatogonia (Figure 1D). In the melanoma metastasis samples CT16 seemed to be mainly cytoplasmic, although in some cells nuclei were also positively stained (Figure 1A).

**Figure 2:**
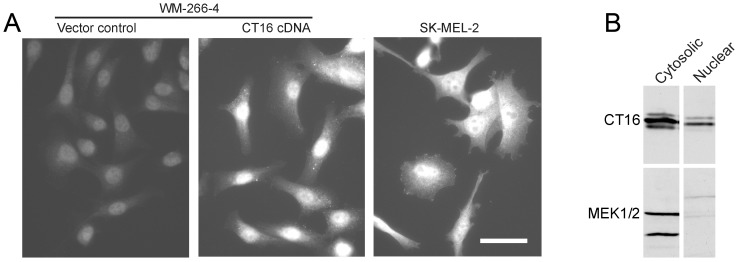
CT16 expression and location in cultured melanoma cells. (A) Images of indicated human melanoma cell lines labeled with rabbit polyclonal antibody against human cancer testis antigen protein CT16 followed by secondary antibody Alexa Fluor 488 goat anti-rabbit IgG (H & L). Images were captured, at equal exposure times to qualitatively compare the expression of the CT16 antigen in the cell lines. Fluorescence intensity in WM-266-4 cells transfected with CT16 cDNA containing plasmid is similar to that of CT16-positive SK-MEL-2 cells. WM-266-4 cells transfected with empty vector shows merely background fluorescence. The scale bar represents 20 µm for all images. (B) Western blot of cytosolic and nuclear fractions of SK-MEL-2 cells. For detection of CT16, the samples were run on a 12% non-denaturing polyacrylamide gel, where CT16 usually migrates as two or three distinct bands. MEK1/2 was detected in the samples run on a 12% denaturing polyacrylamide gel.

The intracellular location of CT16 was studied further in SK-MEL-2 melanoma cells as well as in WM-266-4 melanoma cells stably transfected with CT16 cDNA-containing or intact (control cDNA) containing expression vector. The CT16 expression of the CT16 transfected cells had been verified both at the mRNA and protein level (Figure S2). Immunofluorescence assay using the CT16 antibody (Figure 2A) confirmed the specificity of the antibody, since CT16 negative WM-266-4 cells transfected with the control plasmid showed some background fluorescence only (Figure 2A). In CT16 positive cells CT16 appears to localize both in the nucleus (excluding nucleoli) and cytoplasm. To confirm the localization in the nucleus, SK-MEL-2 nuclear extract was prepared and analyzed by Western blotting. CT16 was found both in the nuclear and cytosolic fraction, whereas a cytosolic control protein MEK1/2 was only present in the cytosolic fraction (Figure 2B).

### CT16 does not affect cell proliferation

**Figure 3:**
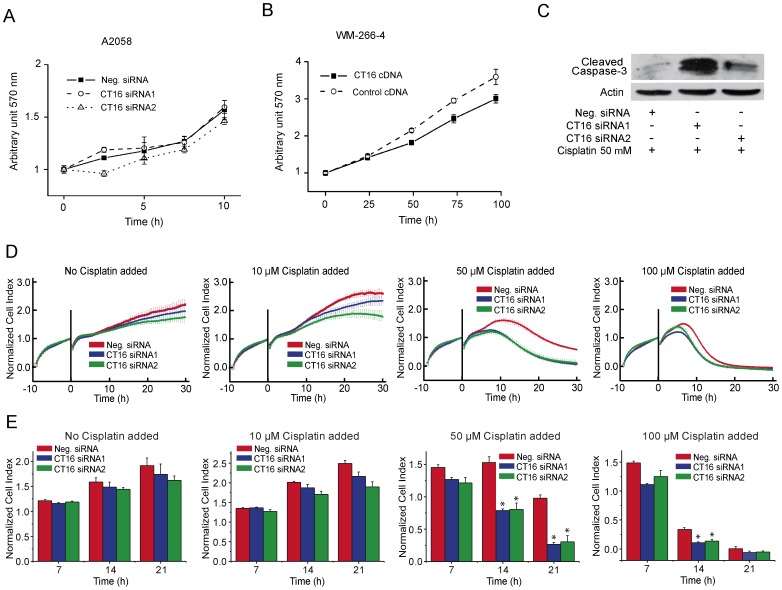
Effect of CT16 on cell proliferation and cisplatin-induced apoptosis. (A) The A2058 cells treated with CT16 specific or control siRNA were subjected to Cell titer 96 proliferation assay. The solubilized formazan dye product was quantified spectrophotometrically at 570 nm. (B) Proliferation of WM-266-4 stably transfected with CT16 or control cDNA. (C) CT16 siRNA transfected A2058 cells were treated with 50 µM cisplatin for 17 hours to induce apoptosis and cell lysates were blotted for cleaved caspase-3. (D) A2058 cells pretreated with indicated siRNA for 48 h were allowed to attach to the xCELLigence plate for 10 h. The cells were exposed to indicated concentrations of cisplatin at the zero time, and the Cell Index was subsequently measured at 10-min intervals for 30 h. The error bars represent standard deviations of three experiments. The cell index of all samples was normalized to 1 at the zero time. (E) Cross-sections at three time points of the xCELLigence experiment. Asterisks indicate significant difference from control siRNA treatment, *P*<0.001 (ANOVA, Tukey's HSD).

The effect of CT16 on the proliferation was studied with the stably CT16 transfected WM-266-4 cells. In addition, two siRNAs against CT16 mRNA were designed and verified to cause more than 80% inhibition of CT16 expression, which lasted at least 96 hours (Figure S3A–C). The siRNAs were considered to be specific for CT16, since they did not regulate the expression of other CT-antigens, e.g. MAGE-1 or GAGE-1 (Figure S3D). Importantly the melanoma cell lines used in this study did not express PAGE2B, the closest homolog to CT16 (Figure S3E). Neither overexpression of CT16 in the WM-266-4 cells nor treatment of A2058 cells with CT16 specific siRNAs had a significant effect on cell proliferation (Figure 3A, B).

### CT16 promotes melanoma cell survival

To test further the biological role of CT16 we exposed melanoma cells to an apoptosis-inducing cancer drug, cisplatin. A2058 cells were first treated either with CT16 specific siRNAs or control siRNA for 48 hours, and after addition of cisplatin the cell cultures were monitored for 30 h in 10-min intervals using xCELLigence technology. In this technology, the cells are grown on a surface containing integrated micro-electrodes, and the electrical impedance between the electrodes caused by the cells is measured. The relative change in measured electrical impedance is expressed as Cell Index (CI). Both cell number and morphology affect the CI, and cell detachment and death are seen as a decrease in CI.

Cisplatin decreased CI of CT16 siRNA treated cells markedly more than that of control siRNA treated cells (Figure 3D, E). The difference was most evident when cisplatin concentration of 50 µM was used. In contrast, CT16 had no effect on the survival in the presence of staurosporine (0 to 600 nM) (not shown). Thus, it appears that CT16 inhibits cisplatin induced but not staurosporine induced death of A2058 melanoma cells.

In the presence of cisplatin CT16 siRNAs could increase the activation of caspase-3 as shown by Western blotting with antibodies that specifically recognize cleaved caspase-3 (Figure 3C). Thus, CT16 could protect the melanoma cells from apoptosis.

### CT16 regulates survival-associated genes

**Figure 4:**
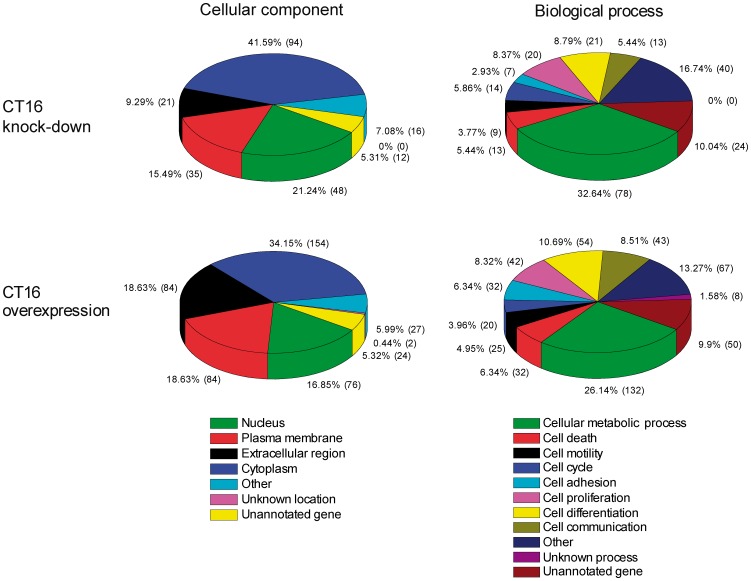
Classification of differentially expressed genes by selected cellular component and biological process gene ontology categories (GO slims). The differentially expressed genes from CT16 knockdown and overexpression experiments were analyzed separately. The number of genes annotated to the particular category is in parenthesis. The mapping to GO slim ontology was done with GoTermMapper (http://go.princeton.edu/cgi-bin/GOTermMapper).

To further examine the role of CT16 in cell survival, two transcriptomic profiling experiments were conducted. In the first experiment, CT16 positive A2058 melanoma cells were treated with CT16 specific siRNA and then compared to A2058 cells treated with control siRNA. In the second experiment, stably control cDNA transfected WM-266-4 melanoma cells were compared to stably CT16 cDNA transfected cells. The microarray data have been deposited, according to MIAME guidelines, in NCBI's Gene Expression Omnibus [25], accession number GSE31352 (http://www.ncbi.nlm.nih.gov/geo/query/acc.cgi?acc=GSE31352). The rank product algorithm was used to select differentially expressed genes [26]. The algorithm detects genes that are consistently found among the most up- or downregulated genes across the replicates. The use of ranks instead of expression values increases the robustness against noise. The genes with rank product false discovery rate (FDR)<0.05 were submitted to gene ontology analysis by GoTermMapper tool that maps the genes to selected cellular component and biological process gene ontology categories (GO slims). The overall distribution by cellular component or biological process of the differentially expressed genes was found to be similar in both CT16 overexpression and knockdown experiments (Figure 4). To identify the biological processes the differentially expressed genes may participate in, a functional annotation analysis was carried out using DAVID tool. Only the terms with Benjamini-Hochberg FDR<0.05 were included in the results. The genes that were upregulated by CT16 overexpression were significantly enriched in the biological processes that promote survival or are related to inflammation, chemical homeostasis as well as response to stimulus and wounding (Table 1). The genes that were downregulated by CT16 overexpression were significantly overrepresented solely in the antigen processing and presentation of peptide or polysaccharide antigen via MHC class II process (Table 1). Analysis of differentially expressed genes in CT16 knockdown experiment did not reveal any biological processes with Benjamini-Hochberg FDR<0.05 (not shown).

**Table 1:** Functional annotation of CT16 up- and downregulated genes.

Category	Biological process	FDR^a^
Genes upregulated by CT16 overexpression	Response to wounding	0.0016
	Taxis, chemotaxis	0.0351
	Cellular chemical homeostasis	0.0407
	Anti-apoptosis	0.0378
	Inflammatory response	0.0494
	Cellular ion homeostasis	0.0485
	Positive regulation of response to stimulus	0.0474
Genes downregulated by CT16 overexpression	Antigen processing and presentation of peptide or polysaccharide antigen via MHC class II	0.0003

a Benjamini-Hochberg false discovery rate.

**Figure 5:**
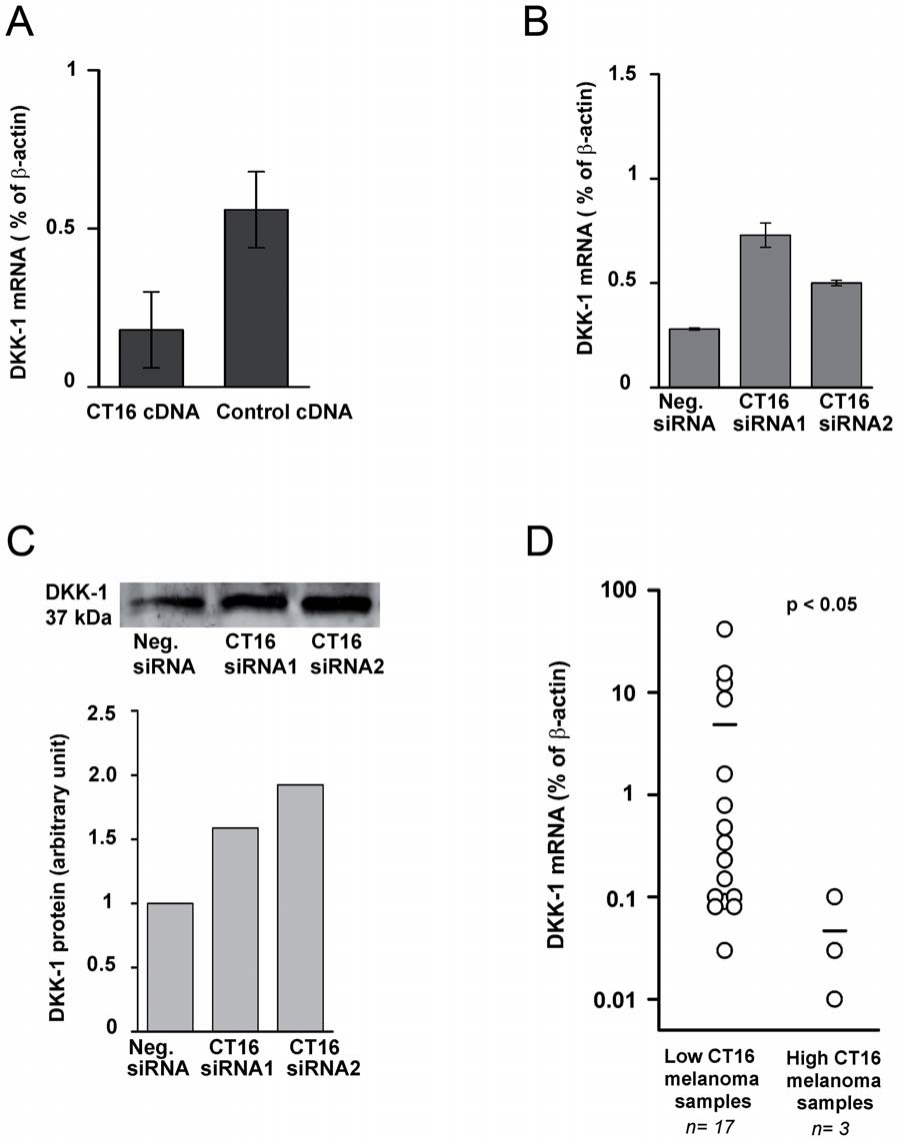
Regulation of DKK1 by CT16. (A) Verification of differential DKK1 mRNA expression in (WM-266-4 cells transfected with control cDNA or CT16 cDNA. by qRT-PCR. (B) Differential DKK1 mRNA expression was also repeatable in A2058 cells treated with indicated siRNAs for 48 h. (C) Western blot for DKK1 in culture medium of A2058 cells treated with indicated siRNAs for 48 h. (D) DKK1 is downregulated in melanoma samples with high CT16 expression. CT16 and DKK1 mRNA levels of melanoma skin metastasis tissue samples were detected by qRT-PCR. The *p* value was obtained by testing the difference in DKK1 mRNA level between the melanoma samples with high CT16 mRNA level (>22% of β-actin mRNA) and those with low CT16 mRNA level (<7% of β-actin mRNA) by Tukey-Kramer test.

To limit the number of genes to be studied, we selected only the genes that were downregulated by CT16 specific siRNA and upregulated due to CT16 overexpression or *vice versa* (rank product FDR<0.1 in both experiments). Of the total of 11 genes fulfilling these criteria, 5 have previously been reported to be involved in cancer progression (Table 2). Based on these experiments CT16 was recognized as a positive regulator of antiapoptotic metallothionein 2A (MT2A) and interleukin 8 (IL8) genes, whereas it inhibited the expression of apoptosis inducing dickkopf 1 (DKK1) gene. In addition, CT16 enhanced the expression of fatty acid binding protein 7 (FABP7), a known promoter of melanoma progression. The differential regulation of FABP7, MT2A and DKK1 genes was confirmed by real-time qRT-PCR analysis of CT16 overexpressing WM-266-4 cells (Figure 5A, Figure S4).

**Table 2:** Genes downregulated by CT16 RNAi in A2058 cells and upregulated by CT16 overexpression in WM-266-4 cells or vice versa.

Symbol	Name	CT16 overexpression^a^	CT16 knockdown^a^	Cancer relevance
DKK1	Dickkopf homolog 1	−2.00	2.81	Activates apoptosis in melanoma and other cancers [27], [28]
CYB5R2	Cytochrome b5 reductase 2	2.81	−1.88	Not known
RAMP1	Receptor (G protein-coupled) activity modifying protein 1	1.75	−1.7	Not known
FABP7	Fatty acid binding protein 7, brain	1.54	−1.63	Melanoma antigen involved in proliferation and invasion [29]
RBPMS2	RNA binding protein with multiple splicing 2	2.86	−1.56	Not known
MT2A	Metallothionein 2A	2.8	−1.53	Protects against ROS-mediated cell death [30]
SERPINA5	Serpin peptidase inhibitor, clade A, member 5	4.04	−1.47	May have a role in breast cancer survival [31]
RRP7A	Ribosomal RNA processing 7 homolog A (S. cerevisiae)	1.78	−1.42	Not known
IL8	Interleukin 8	3.71	−1.42	Inhibits TRAIL- and drug-induced apoptosis of prostate cancer cells [32]. Increases tumorigenicity and metastatic potential of melanoma cells [33]
CDC14B	CDC14 cell division cycle 14 homolog B (S. cerevisiae)	1.89	−1.38	Not known
ARPC1B	Actin related protein 2/3 complex, subunit 1B, 41 kDa	1.73	−1.34	Not known

a Fold change compared to control.

To test the selection criteria of the genes in Table 2, we used real-time qRT-PCR to analyze three genes, CTSL, DDAH1 and BIRC5 that were downregulated by CT16 specific siRNA and upregulated due to CT16 overexpression or *vice versa* but had the rank product FDR>0.1. In these measurements the effect of CT16 on expression could not be confirmed (Figure S4). Thus, the selected rank product FDR threshold value efficiently filtered out false positives, while retaining the true ones.

### CT16 is a negative regulator of DKK1 in melanoma

The extracellular Wnt antagonist DKK1 was chosen for further studies, since it has previously been shown to activate apoptosis and to be downregulated in melanoma and other cancers [27], [28]. Two independent CT16 siRNAs increased DKK1 mRNA levels in A2058 cells (Figure 5B). The effect of CT16 on DKK1 expression was also confirmed at protein level in cell culture medium of A2058 cells by Western blots. Knockdown by CT16 specific siRNA resulted in an increase in the accumulation of DKK1 protein into the medium (Figure 5C).

To find evidence that CT16 also regulates DKK1 expression levels in tumors, 20 melanoma skin metastasis samples were analyzed by real-time qRT-PCR. The CT16 mRNA level relative to β-actin was below 7% in 17 samples that were grouped as low CT16 melanoma samples. CT16 mRNA level was above 22% of β-actin in three samples that were grouped as high CT16 melanoma samples. Interestingly, the high CT16 samples had significantly (p<0.05, Tukey-Kramer test) lower average DKK1 mRNA level compared to that of low CT16 samples (Figure 5D), which suggests that high CT16 level may be one reason for previously described downregulation of DKK1 expression in melanoma.

### CT16 regulates its target genes in a p53 and DNA methylation independent manner

The molecular interactions of X-CTAs are only known in few cases. Since MAGE proteins are known to affect p53 functions (Yang et al., 2007), we wanted to study the effects in different CT16 positive cell lines. No change in p53 protein expression was detected by immunoblotting in CT16 siRNA knockdown SK-MEL-2 cells compared to the control siRNA treated cells (Figure S5A). Similar result was also received between the CT16 and control cDNA transfected WM-266-4 cells (Figure S5B). We also tested the effects of CT16 in human osteosarcoma Saos-2 cells that are known to have no p53 expression. The negative expression of p53 in Saos-2 cells was confirmed with qRT-PCR (not shown). The result showed, similarly as in the A2058 cells, a clear upregulation, of DKK1 after CT16 knockdown, indicating that p53 is not involved in the process (Figure S5C). Neither had CT16 any effect on the conformational change of p53 in the crude extract of WM-266-4 cells (Figure S5D). Furthermore, there was no difference in the p53 stability between the CT16 siRNA treated A2058 cells and the control cells after inhibition of protein synthesis with CHX Figure S5E). In accordance with that, no significant enrichment of p53 target genes among differentially expressed genes was found (not shown). Together these results support the idea that p53 do not mediate the effects of CT16 or participate in apoptosis.

Some X-CTAs also affect gene methylation and post-translational modifications in histones. To study this, a genome-wide promoter-specific chromatin immunoprecipitation analysis for WM-266-4 cells stably transfected with CT16 or control cDNA was carried out using DNA methylation specific and acetylated histone H3 specific antibodies and a microarray covering 30,000 human promoters. The sample-specific genes, defined as those with peaks (detected and evaluated by NimbleScan software) having FDR<0.05 in the sample and FDR≥0.2 in the control or vice versa, were analyzed by using DAVID. There were no significantly enriched genes in any gene ontology categories among the sample-specific genes (not shown). We also compared the lists of differentially regulated genes in the WM-266-4 derived cell lines with the sample-specific gene-lists from both methylation specific and histone acetylation specific immunoprecipitation analyses (Table S1). The results suggest there is a link between histone H3 acetylation and gene expression of CT16 regulated genes. However, it is unclear whether CT16 is involved in histone acetylation events or the result merely reflects the well-known connection between histone H3 acetylation and gene expression. In contrast to histone H3 acetylation, DNA methylation showed no correlation with CT16 regulated genes suggesting that the changes in gene expression profile by CT16 are not mediated by DNA methylation.

Recently, it has been shown that class I MAGE proteins regulate KAP1 and KRAB domain zinc finger transcription factor-mediated gene repression via interaction with KAP1 [34]. However, it is not very likely that also CT16 would interact with KAP1, since the KAP1 targets identified in a genome-wide chromatin immunoprecipitation analysis of KAP1 binding [35] were not enriched among differentially expressed genes from either the CT16 knockdown or overexpression experiments (not shown).

We have previously excluded the possibility that CT16 could directly bind to GC-rich sequences in double-stranded DNA [19] in a similar manner as its paralog PAGE4 [18]. Here, we could not show localization of CT16 to the immediate promoters of its target genes (Figure S6). Thus, the mechanism of CT16 action, like the function of most other X-CTAs, remains unknown.

## Discussion

The effective treatment of metastatic melanoma is a major unsolved problem. Therefore, there is an urgent need to get new information about molecular mechanisms that regulate survival and drug resistance in melanoma. Recent results from clinical trials have generated optimism that in the near future new specific drugs, such as ipilimumab [36] and BRAF inhibitors [37], could be used to stop the progression of an advanced disease. However, many melanomas also seem to develop resistance against the new drug molecules quickly, sometimes in months [38].

We have recently reported that the expression of CT16 is frequently induced in human melanoma. Furthermore, serum CT16 can be measured and used to follow disease progression [16]. Here we show that CT16 protects melanoma cells against cisplatin (50 µM) induced cell death. Platinum complexes bind to and crosslink DNA, which leads to apoptosis. Interestingly, CT16 could not prevent staurosporine-induced cell death. Staurosporine is an alkaloid that inhibits a wide spectrum of protein kinases [39], and thus may activate apoptosis by several different mechanisms [40]. The cellular functions of most CTAs are unknown, but in addition to CT16 also certain other X-CTAs have been linked to cell survival. Transfection of ovarian cancer cells by MAGE-A2 and MAGE-A6 can induce paclitaxel and doxorubicin resistance by an unknown mechanism [41]. Furthermore, GAGE-7 has been described to act as an anti-apoptotic protein in HeLa cells [17], [42]. Most recently, a prostate cancer associated antigen, PAGE4, was reported to inhibit stress-induced cell death [18].

Based on our genome wide gene expression analysis, CT16 can negatively regulate specific biological processes such as apoptosis and antigen presentation to enhance survival of cancer cell. The mechanism of CT16 action seems to be at least partly related to the fact that it can regulate the expression of apoptotic and antiapoptotic genes, such as metallothionein 2A, IL-8 and DKK1. These three genes were among the eleven genes that were found to be regulated by CT16 both in the CT16 silencing and overexpression experiments. Metallothionein 2A is known to protect HeLa cells against ROS-induced death [30], whereas IL-8 inhibits TRAIL- and drug-induced apoptosis of prostate cancer cells [33] and increases tumorigenicity and metastatic potential of melanoma cells [32]. Most interestingly, CT16 was found to be a negative regulator of DKK1, a gene described to be significantly downregulated in malignant melanoma [43]. DKK1 is an extracellular inhibitor of anti-apoptotic Wnt-signaling pathway [44] that can induce apoptosis in melanoma cells [27]. Therefore, decrease in DKK1 expression is considered to be an important survival mechanism in melanoma cells. When we analyzed 20 melanoma skin metastases in their DKK1 and CT16 mRNA expression, three samples showed exceptionally high CT16 levels and concomitantly very low DKK1 expression. Thus, our data suggest that CT16 overexpression is one, but probably not the only mechanism that leads to DKK1 suppression in melanoma. CT16 also enhances the expression of fatty acid binding protein 7 (FABP7), a known promoter of melanoma progression [29].

The exact mechanism of how CT16 regulates the expression of these genes remains to be studied. PAGE4, a paralog of CT16, was recently shown to bind directly to DNA [18]. Furthermore, both CT16 and PAGE4 are intrinsically disordered proteins [18], [19], [45]. However, CT16 binding to DNA has not been observed [19]. Therefore, despite the homology between PAGE4 and CT16 the mechanism of action can differ markedly between these two proteins. Furthermore, we have excluded the two other known mechanisms of CTA-X action. Our data indicate that p53 does not mediate the effects of CT16 despite the fact that MAGE proteins acts in this manner. Neither could we detect any connection between DNA methylation and CT16 regulated genes.

To conclude, recent progress in the development of drug therapy against advanced melanoma has led to urgent need to develop new biomarker of disease progression. We have recently suggested that serum CT16 levels could be used to monitor the efficacy of the treatment and putative relapses. Here, we report that CT16 also contributes to the pathogenesis of the disease by regulating both apoptotic and antiapoptotic genes and promoting melanoma cell survival. Therefore, CT16 is a putative target molecule for drug development aimed to potentiate the effect of apoptosis inducing drugs.

## Materials and Methods

### Ethics statement

The clinical studies were approved by the institutional review board of Turku University Hospital, and each patient provided written informed consent to participate in the trials and/or to allow his/her tissue samples to be used for research purposes.

### Cell lines and transfections

The melanoma cell lines A2058, SK-MEL-2, and WM-266-4 originally obtained from American Type Culture Collection (Manassas, VA) were authenticated by Health Protection Agency Culture Collections (Porton Down, UK) using STR analysis method. The melanoma cell lines were maintained at 5% CO_2_ and 37°C in Dulbecco's Modified Eagle Medium (DMEM) (Invitrogen, Carlsbad, CA) supplemented with 10% fetal bovine serum. The CT16 cDNA including the stop codon was cloned by PCR from the expression vector for CT16 [16] and inserted into *Xba*I and *Eco*RI sites of pEXPR-IBA103 plasmid (IBA GmbH, Göttingen, Germany). An amount of 1 µg of the CT16 cDNA-containing or intact (control cDNA) pEXPR-IBA103 vector was transfected into WM-266-4 cells using Fugene 6 transfection reagent (Roche, Basel, Switzerland) according to the manufacturer's instructions. After incubation for 48 h the G418-resistant cells were selected by 0.8 mg/ml G418 (Invitrogen). The stably transfected cells were maintained in medium containing 0.2 mg/ml G418. The p53 negative human osteosarcoma cell line Saos-2 was used to study the DKK-1 mRNA expression in a p53 independent manner. Saos-2 cells were cultured similarly as the melanoma cell lines.

### Tissue samples

The human melanoma tissue samples were obtained during normal surgical proceedings done for diagnosis or treatment of metastatic melanoma. Half of the removed samples were cut and put into liquid nitrogen and stored at −80°C while the other half was formalin-fixed for pathological diagnosis and immunohistochemical purposes. An amount of 150 mg of each sample was cut to sections while still frozen and put on glass slide for immunohistochemical analysis or used for RNA isolation.

### siRNA transfection

The A2058 and SK-MEL-2 cells were transfected with CT16 targeting or non-targeting control siRNAs (Eurogentec, Liege, Belgium) at a confluence of 50–70% using SiLentFect Lipid reagent (Bio-Rad laboratories, Hercules, CA) according to the manufacturer's recommendations. The cells were incubated for 48, 72 and 96 hours. The sequences of the CT16 siRNAs and non-silencing control were as follows: (CT16 siRNA1) 5′-GCUGGAAAUUUGACUGCUAdTdT, (CT16 siRNA 2) 5′-GGGACUGAUGUGGAAGCUUdTdT and (control) 5′-AAGAUUCGUAACCGUUGUGdTdT.

### Cell proliferation assay

Stably CT16- and control-cDNA transfected WM-266-4 cells (2500 cells/well) and A2058 cells (10000 cells/well) incubated with CT16 siRNA for 48 h were seeded and cultured O/N on 96-well plates at 5% CO2 and 37°C. Six parallel samples were used. The cell proliferation assay was started the following day by using Cell titer 96 nonradioactive cell proliferation assay kit (Promega, Madison, WI) according to the manufacturer's protocol. The solubilized formazan dye product was quantified spectrophotometrically at 570 nm with a 96 well plate reader.

### Real-time monitoring of cells

The siRNA-transfected A2058 melanoma cells were detached from the culture dish plate by trypsin, collected by centrifugation at 500× g for 5 min at room temperature, and resuspended in DMEM. After measuring the background by the xCELLigence real-time cell electronic analyzer (Roche) using 50 µl DMEM per well in a 96-well xCELLigence plate, 10^4^ cells in 100 µl of DMEM were added to each well. The plate was transferred to the cell incubator for 30 min (37°C, 5% CO_2_), and then transferred back to the xCELLigence system to start the run. Impedance was measured every 10 min for 10 h, after which the plate was taken out from the system for drug addition. Culture medium was discarded, and varying amounts of cisplatin (Sigma-Aldrich, St. Louis, MO) in 100 µl of DMEM were added to induce apoptosis in the melanoma cells. The plate was transferred back to the system, and monitoring was continued for 30 h. Impedance was measured as Cell Index (CI) values which were normalized.

### Real-time quantitative RT-PCR

Total RNA isolation, cDNA synthesis and real-time quantitative RT-PCR (qRT-PCR) were performed as described [46]. The primers and probes for CT16 have been described previously [16]. DKK1, CTSL and β-actin probes were labeled with 6-carboxyfluorescein (FAM) and 6-carboxy-tetramethyl-rhodamine (TAMRA). The primers and probe were designed using Primer Express v.1.5 software (Applied Biosystems, Pleasanton, CA). The self-designed primers and probes are listed in Table S2. The DDAH1, BIRC5, FABP7 and MT2A gene primers and probes were synthesized/ordered as gene expression assay mix (20×) consisting of forward and reverse primers and MGB probe (FAM dye-labeled) (Applied Biosystems). The qRT-PCR was run by 7900HT Fast Sequence Detection System (Applied Biosystems) and the cycle threshold values were determined by SDS 2.3 and RQ manager 1.2 software (Applied Biosystems).

### Western blotting

The Western blotting for CT16 was done as described [16]. For detection of DKK1 in the culture medium, the proteins from 2 ml of the cultured cell media were concentrated with centricon centrifugal filter, YM-10 (Millipore, Billerica, MA) by centrifuging at 5000× g at 4°C until a retentate volume of approximately 60 µl was generated. Equal amounts of protein were separated in a 10% polyacrylamide gel and transferred to nitrocellulose membrane (PerkinElmer). After blocking with 5% nonfat dry milk, 1% BSA and 0.1% Tween in TBS, DKK-1 antibody (dilution 1∶500, sc-25516, Santa Cruz Biotechnology, Santa Cruz, CA) was applied overnight at 4°C. DKK-1 antibody was detected with HRP-conjugated goat antirabbit IgG (dilution 1∶5000, sc-2054, Santa Cruz Biotechnology) and SuperSignal West Pico chemiluminescent substrate (Thermo Fisher Scientific, Waltham MA). Western blotting of p53 was performed from cell lysates on 10% polyacrylamide gel and detected with mouse monoclonal p53 antibody (dilution 1∶500, sc-126, Santa Cruz Biotechnology) and HRP-conjugated goat-antimouse IgG (dilution 1∶5000, sc-2005, Santa Cruz Biotechnology). CT16 siRNA transfected A2058 cells (48 hours) were treated with 50 µM Cisplatin for 17 hours to induce apoptosis. The cells were lysed and cleaved caspase-3 was detected with polyclonal cleaved caspase-3 antibody (dilution 1∶500, Asp-175, 9661, Cell Signaling, Technology, Beverly, MA) and HRP conjugated antirabbit IgG (dilution 1∶5000). MAGE-1 and GAGE-1 in A2058 cells treated with siRNA for 48 hours were detected with mouse monoclonal MAGE-1 antibody (dilution 1∶250, 6C1, Thermo Fischer Scientific) and mouse polyclonal GAGE-1 antibody (dilution 1∶500, ab67412, Abcam, Cambridge, UK), respectively. The cell lysates used for immunoblotting of cleaved caspase-3, MAGE-1 and GAGE-1 were run on a 12% polyacrylamide gel.

### p53 conformation analysis

The WM-266-4 cells were lysed in 1% Triton X-100, 1 mM EDTA, 10 mM Tris/HCl, pH 7.4 supplemented with protease inhibitors (10 mg/l aprotinin, 10 mg/l leupeptin, 2 mM PMSF) and phosphatase inhibitors (1 mM Na_3_VO_4_, 10 mM NaF, 10 mM Na_4_P_2_O_7_). The lysate was centrifuged at 4°C at 13,000×g for 15 min, and the supernatant was incubated at 37°C for 0, 30, 60 or 120 min in the presence of 50 µg/ml recombinant glutathione S-transferase (GST) or CT16. The inactive and active p53 were immunoprecipitated with PAb240 (Invitrogen) and PAb1620 (Abcam) respectively, and p53 was detected by western blotting as described [47] except that p53 DO-1 antibody (sc-126, Santa Cruz Biotechnology) was used.

### p53 stability assay

A2058 cells were treated with CT16 siRNA1, CT16 siRNA2 or control siRNA for 48 hours followed by addition of protein synthesis inhibitor cycloheximide (CHX) (Sigma-Aldrich) to a final concentration of 30 µg/ml. Cells were washed and lysed after 0, 3 and 6 hours after CHX-addition, and cell lysates (60 µg) were immunoblotted for total p53 .

### Gene promoter and transcription assays

Two promoter-reporter fusion constructs, one with a DKK1 promoter region from −1037 to +163 (1200 bp), and the other with a region from −1056 to +155 (1211 bp) were used to measure the activity of the DKK1 promoter. pGL3-DKK1 (with 1200 bp region) was a kindly donated gift from Dr. Tetsu Akiyama [48], The 1211-bp promoter region was synthesized by Life Technologies (Carlsbad, CA) and cloned into pGL3-Basic plasmid (Promega) using *Kpn*I and *Hin*dIII sites to get the plasmid pGL3-DKK1CN. CT16 siRNA transfected A2058 cells were used for measurement of luciferase activity. Around 30,000 cells/well were plated in 24-well plates and on the following day the cells were transfected either with 200 ng of pGL3-DKK1 or 200 ng pGL3-DKK1CN and cotransfected with 100 ng of pRL-SV40 (Promega). Two days after transfection the measurement of luciferase activity was performed by using dual luciferase reporter assay system (Promega) according to the manufacturer's protocol. The luminescent signal from the firefly luciferase (pGL3-DKK1 or pGL3-DKK1CN) was normalized with the signal from the *Renilla* luciferase (pRL-SV40).

The CT16 specific chromatin immunoprecipitation assay was performed with EZ-Magna-ChIP A/G kit (Millipore) and the CT16 specific antibody according to the manufacturer's instructions. The enrichment of promoters was assessed by a standard PCR assay.

### Preparation and analysis of nuclear and cytosolic fractions of SK-MEL-2 cells

The SK-MEL-2 cells from culture plate were trypsinized and suspended in homogenization buffer (50 mM Tris/HCl pH 7.4, 7.5% glycerol, 0.5 mM dithiotreitol, 150 mM NaCl, 1 mM EDTA, 2 mM MgCl_2_) supplemented with one Complete mini protease inhibitor tablet, EDTA-free (Roche) per 10 ml of buffer and homogenized with Dounce homogenizer (Sigma-Aldrich). The homogenate was centrifuged at 750× g for 10 min at 4°C. The supernatant was further centrifuged at 20 000× g for 1 h at 4°C. This supernatant containing the cytosolic proteins was saved for further analysis. The pellet obtained from the homogenate centrifugation containing the nuclei was resuspended in 10 mM HEPES, 10 mM, KCl, 0.25 M sucrose, 10 mM MgCl_2_, pH 7.9. For purification of the nuclei, the suspension was layered on equal volume of 10 mM HEPES, 10 mM, KCl, 0.35 M sucrose, 0.5 mM MgCl_2_, pH 7.9 and centrifuged at 1400× g for 5 min. The resuspension and centrifugation were repeated, and the pellet was resuspended in lysis buffer (50 mM Tris-HCl, 7.5% (v/v) glycerol, 150 mM NaCl, 1 mM EDTA, 2 mM MgCl_2)_ supplemented with one Complete mini protease inhibitor tablet, EDTA-free per 10 ml of buffer. The nuclear proteins were extracted by sonication and centrifugation at 18 000× g for 10 min at 4°C. Equal amounts of either nuclear or cytosolic proteins were run on a non-denaturing polyacrylamide gel (12%) for CT16 detection and on a sodium dodecylsulphate polyacrylamide gel (12%) for MEK1/2 detection. Western blotting was done as described previously [16] except that MEK1/2 was detected with MEK1/2 Antibody #9122 (Cell Signaling).

### Transcriptome analysis

Total RNA triplets extracted with TRIzol reagent (Invitrogen) from the A2058 cells treated with CT16 siRNA1 or control siRNA for 48 h and WM-266-4 cells stably transfected with CT16 cDNA- containing or intact vector were prepared and analyzed for genome wide screening at the Finnish Microarray and Sequencing Centre (FMSC), Turku, Finland. The amplified and biotinylated RNA was hybridized on Illumina's Sentrix Human-6 Expression BeadChips and scanned with Illumina BeadArray Reader, BeadScan software v3.5.49 (Illumina Inc., San Diego, CA).

The microarray data were normalized using the quantile normalization method in Bioconductor (http://www.bioconductor.org), and differentially expressed genes were identified using the rank product algorithm (Bioconductor RankProd package).

### Histone H3 acetylation and DNA-methylation specific immunoprecipitation analyses on microarrays

Acetyl-Histone H3 specific and methylated DNA specific immunoprecipitations were performed with Upstate Acetyl-Histone H3 Immunoprecipitation (ChIP) Assay Kit (Millipore) and MeDIP kit (Diagenode, Liege, Belgium) according to manufacturers' instructions, respectively. The immunoprecipitates and the input DNA samples were amplified with WGA2 kit (Sigma), subsequently purified by GenElute PCR Cleanup kit (Sigma) and analyzed at Nimblegen services using HG18 Deluxe Promoter HX1 array and NimbleScan software (Roche).

### Immunohistochemical analysis of CT16

The sections from frozen tissue samples were fixed with acetone, and the endogenous peroxidase was blocked with 0.3% H_2_O_2_ in TBS for 20 min followed by washing with TBS three times for 5 min. To reduce non-specific staining, the sections were incubated with goat serum diluted 1∶66 in TBS at RT for 90 min. CT16 was detected with 1∶10,000 dilution of ProteinG-affinity purified CT16-specific antibody [16] in TBS, and the primary antibody was detected by Vectastain ABC kit (Vectorlabs, Burlingame, CA) according to the manufacturer's instructions. The sections were stained with DAB staining solution (0.05% diaminobenzidine, 10 mM imidazole and 0.01% H_2_O_2_ in TBS), counterstained with Mayer's hematoxylin and mounted.

Microphotographs were taken using a Leica DM3000 microscope, Leica DFC420 digital camera and Leica Application Suite version 2.8.1 software (Leica Microsystems GmbH, Wetzlar, Germany). Adobe Photoshop CS4 and ImageJ (Rasband, W.S., ImageJ, U. S. National Institutes of Health, Bethesda, Maryland, USA, http://imagej.nih.gov/ij/, 1997–2011) were used to analyze staining intensities.

### CT16 Immunofluorescence

SK-MEL-2 cells and WM-266-4 cells transfected with either CT16 cDNA- containing or intact vector were seeded on glass coverslips at a concentration of 20,000 cells/cm^2^ and incubated overnight. The cells were labeled by indirect immunolabeling method as described [49] using CT16-specific antibody at a concentration of 15 µg/ml as a primary antibody and Alexa Fluor 488 goat anti-rabbit IgG (H & L) (Invitrogen) at a dilution of 1∶100 as a secondary antibody. All samples were imaged with a Zeiss Axioscope II (Carl Zeiss, Jena, Germany). The Alexa Fluor 488 labeled samples were imaged using the EGPF filter. Images were captured, at equal exposure times, with a QImaging QICam digital camera (QImaging, Surrey, British Columbia, Canada) using Northern Eclipse Software (Empix Imaging, Mississauga, Ontario, Canada) and saved as 8-bit grayscale images in TIFF file format.

### Data analysis

Microsoft Access was used to compare the gene lists and to identify the sample-specific genes. The gene lists were mapped to selected cellular component and biological process gene ontology categories (GO slims) by using GoTermMapper (http://go.princeton.edu/cgi-bin/GOTermMapper). Assigning the gene lists to a set of biological process gene ontology categories where the broadest terms have been filtered out (GO Fat) was done by using DAVID Functional Annotation Tool [50], [51]. The Pearson correlation analysis (IBM SPSS Statistics version 16.0) was used to calculate the correlation coefficient r between CT16 mRNA level and immunohistochemical staining. The Tukey-Kramer test (SAS 9.2, SAS Institute Inc., Cary, NC) was applied to evaluate the statistical significance of the difference in DKK1 expression between the groups with high and low CT16 mRNA level of human melanoma skin metastasis samples.

## Supporting Information

Figure S1 Correlation between mRNA level and CT16 specific staining in the melanoma samples. The brown areas in the images of the immunohistochemically stained melanoma samples were extracted and transformed into negative grayscale images, which were quantitated. The lightness of a pixel is expressed as the grey value from 0 to 255, with 0 being black and 255 being white. The mean gray values (the sum of the gray values of all the pixels in the image divided by the number of pixels) were plotted against CT16 mRNA-level of CT16 of the corresponding samples. The two-tailed p value for significance of Pearson correlation is shown.
(TIF)

Figure S2 Verification of CT16 expression in melanoma cells stably transfected with CT16. (A) CT16 mRNA expression measured by qRT-PCR analysis in WM-266-4 melanoma cells stably transfected with a plasmid containing CT16 cDNA. SK-MEL-2 and A2058 melanoma cells were used as positive controls, and the wild type WM-266-4 cells or WM-266-4 cells stably transfected with control cDNA were used as negative controls. (B) Western blot for CT16 of the same cell lines as above. Equal amounts of total protein from the corresponding cell lines were separated on a native polyacrylamide gel. Recombinant CT16 was used as a standard (CT16 rec.).
(TIF)

Figure S3 Verification of CT16 silencing. (A) CT16 was successfully down regulated at mRNA level after 48–96 hours incubation of CT16 siRNA1 in the melanoma cell line Sk-mel-2. (B) Corresponding protein levels after CT16 knockdown by CT16 siRNA1 and 2. (C) CT16 mRNA silencing in melanoma cell line A2058 with a 48–96 hours incubation of CT16 siRNA1 and 2. (D) Equal amounts of MAGE-1 and GAGE-1 were detected by immunoblotting of lysates from CT16 siRNA (48 hours) transfected A2058 cells. (E) No detection of PAGE2B in the indicated melanoma cell lines with RT-PCR. cDNA of normal testis was used as a positive control for PAGE2B.
(TIF)

Figure S4 Verification of the effect CT16 overexpression on selected genes. The effect of CT16 overexpression on the mRNA levels of FABP7 and MT2A, which had the rank product FDR value<0.01 could be verified by qRT-PCR while the differential expression of CTSL, DDAH1 and BIRC5 (FDR>0.01) was not repeatable.
(TIF)

Figure S5 Testing the effect of CT16 on p53. (A) Sk-mel-2 cells were treated with CT16 siRNAs for 48 hours and immunoblotted for total p53 expression. (B) Western blot analysis of total p53 expression in stably transfected WM-266-4 cells, expressing CT16, and control cDNA cells, expressing empty vector. (C) DKK-1 mRNA expression was analyzed with qRT-PCR in CT16-siRNA (48 hours) transfected Saos-2 cells. Saos-2 are p53- negative cells. (D) The cell lysate from WM-266-4 cells was incubated at 37°C in the presence of either CT16 or G8T (50 µg/ml) for indicated periods of time. After incubation, the active and inactive p53 were immunoprecipitated with conformation specific antibodies, and the immunoprecipitates were analyzed by immunoblotting with p53 specific antibody. (E) A2058 cells were treated with CT16 siRNAs for 48 hours, followed by addition of 30 µg/ml CHX. After addition, cells were lysed at indicated timepoints and immunoblotted.
(TIF)

Figure S6 The influence between CT16 and promoters of CT16-regulated genes. (A) No interaction between CT16 and the indicated promoters of CT16-regulated genes in A2058 cells detected by chromatin immunoprecipitation. Anti-RNA Polymerase was served as a positive control. (B, C) A2058 cells transfected with either pGL3-DKK1 or pGL3-DKK1CN, gave no significant change in the relative luciferase activity between neg. siRNA and the CT16 siRNA samples. The error bars represent standard deviations from three independent experiments.
(TIF)

Table S1 Comparison of chromatin immunoprecipitation data with differentially regulated genes in transfected WM-266-4 cells.
(PDF)

Table S2 Primers and probes designed for qRT-PCR.
(PDF)
