# Supplementary figures and images for: Melanoma-Associated Cancer-Testis Antigen 16 (CT16) Regulates the Expression of Apoptotic and Antiapoptotic Genes and Promotes Cell Survival
Source: PLoS One. 2012 Sep 21;7(9):e45382. doi: 10.1371/journal.pone.0045382 (PMC3448647; doi:10.1371/journal.pone.0045382)

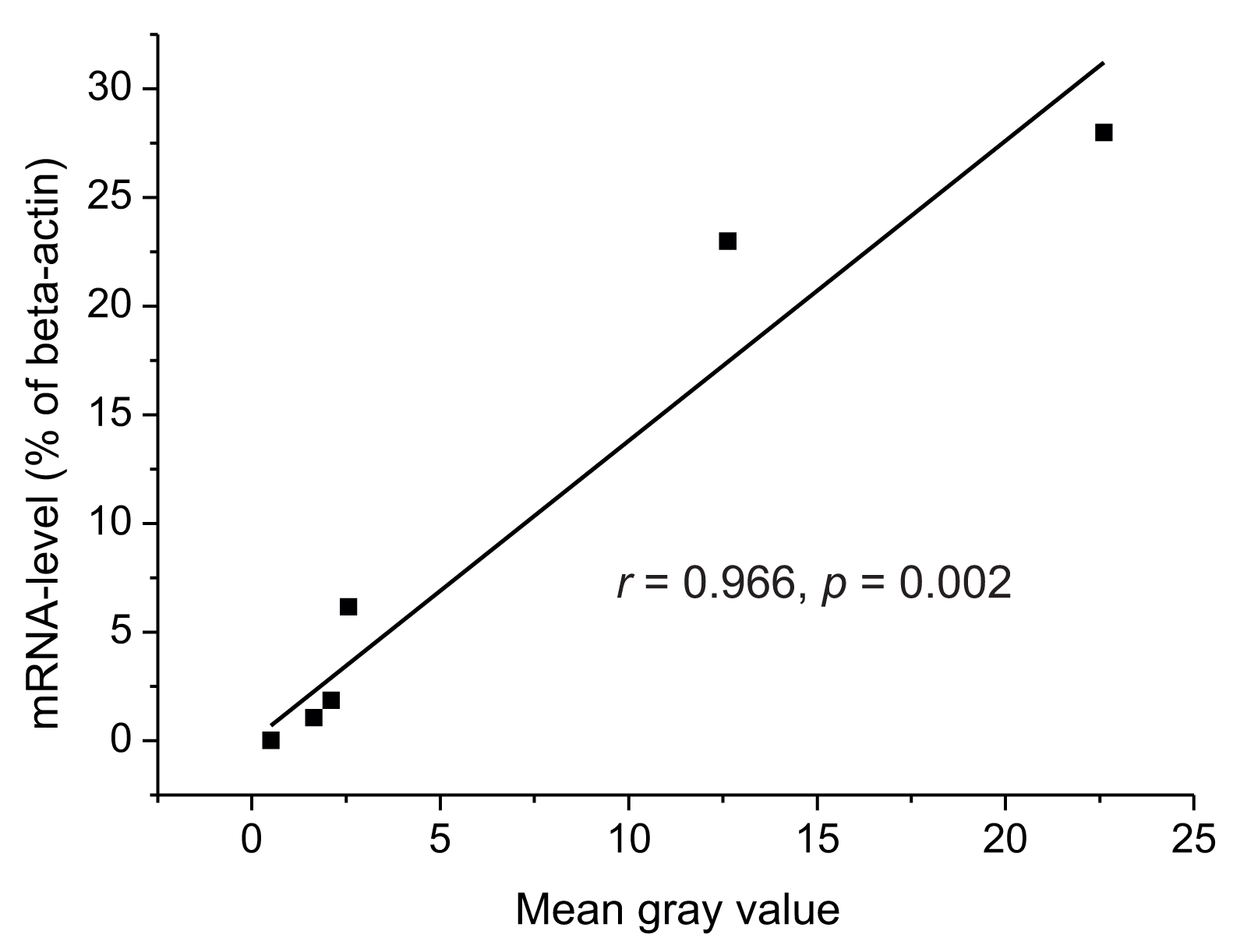

Supplement: Figure S1 — Correlation between mRNA level and CT16 specific staining in the melanoma samples. The brown areas in the images of the immunohistochemically stained melanoma samples were extracted and transformed into negative grayscale images, which were quantitated. The lightness of a pixel is expressed as the grey value from 0 to 255, with 0 being black and 255 being white. The mean gray values (the sum of the gray values of all the pixels in the image divided by the number of pixels) were plotted against CT16 mRNA-level of CT16 of the corresponding samples. The two-tailed p value for significance of Pearson correlation is shown. (TIF) [file pone.0045382.s001.tif]

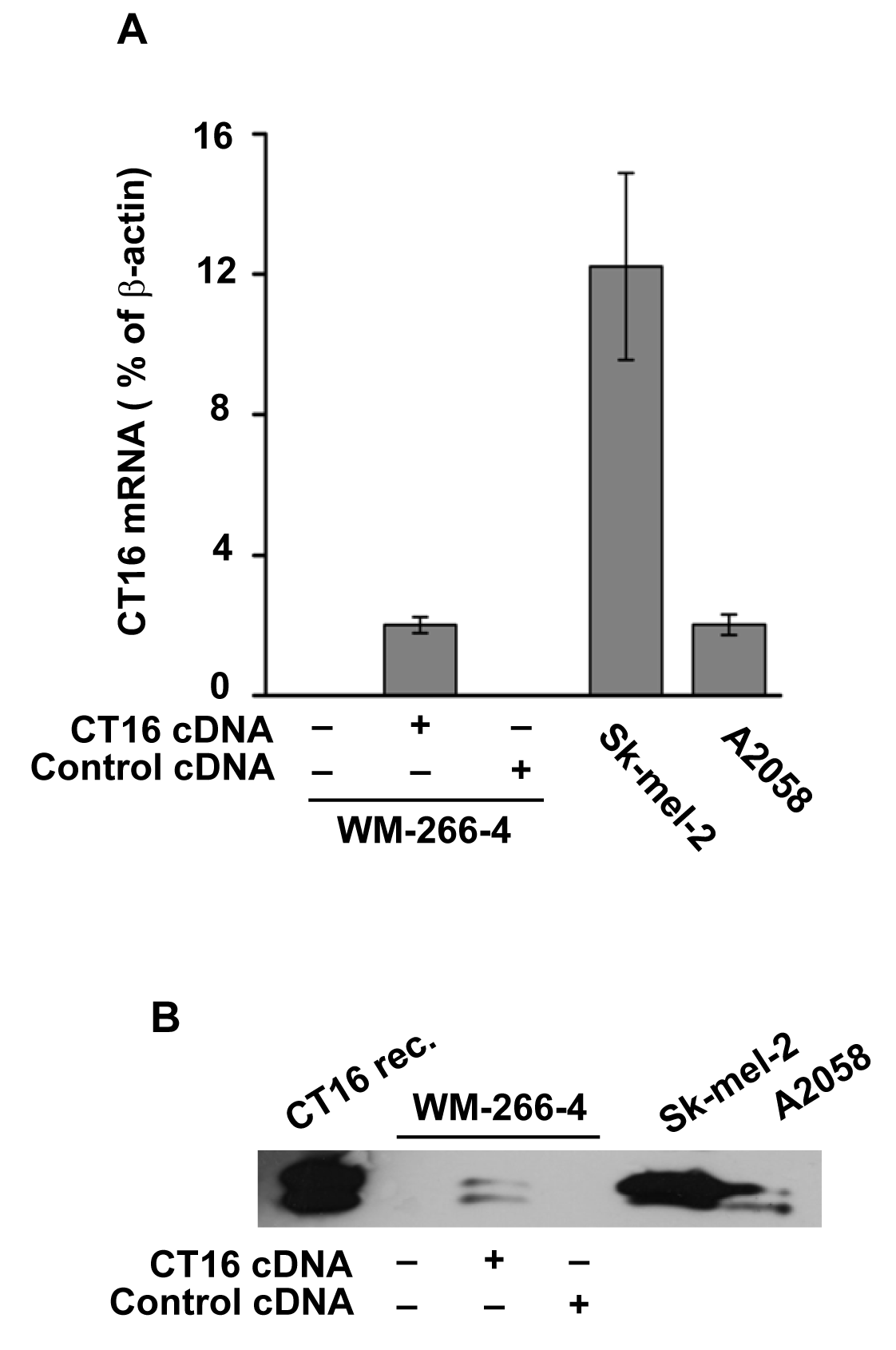

Supplement: Figure S2 — Verification of CT16 expression in melanoma cells stably transfected with CT16. (A) CT16 mRNA expression measured by qRT-PCR analysis in WM-266-4 melanoma cells stably transfected with a plasmid containing CT16 cDNA. SK-MEL-2 and A2058 melanoma cells were used as positive controls, and the wild type WM-266-4 cells or WM-266-4 cells stably transfected with control cDNA were used as negative controls. (B) Western blot for CT16 of the same cell lines as above. Equal amounts of total protein from the corresponding cell lines were separated on a native polyacrylamide gel. Recombinant CT16 was used as a standard (CT16 rec.). (TIF) [file pone.0045382.s002.tif]

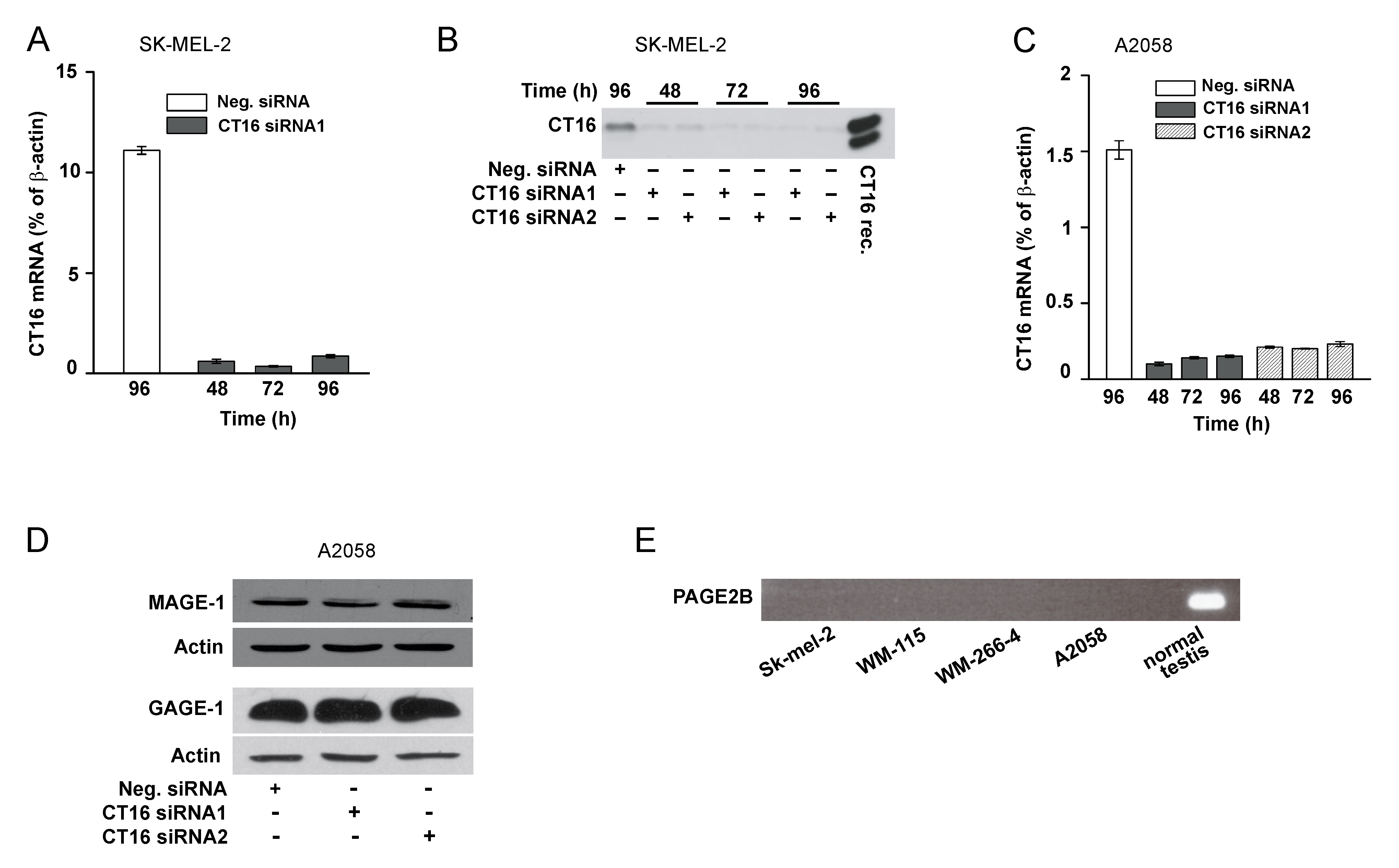

Supplement: Figure S3 — Verification of CT16 silencing. (A) CT16 was successfully down regulated at mRNA level after 48–96 hours incubation of CT16 siRNA1 in the melanoma cell line Sk-mel-2. (B) Corresponding protein levels after CT16 knockdown by CT16 siRNA1 and 2. (C) CT16 mRNA silencing in melanoma cell line A2058 with a 48–96 hours incubation of CT16 siRNA1 and 2. (D) Equal amounts of MAGE-1 and GAGE-1 were detected by immunoblotting of lysates from CT16 siRNA (48 hours) transfected A2058 cells. (E) No detection of PAGE2B in the indicated melanoma cell lines with RT-PCR. cDNA of normal testis was used as a positive control for PAGE2B. (TIF) [file pone.0045382.s003.tif]

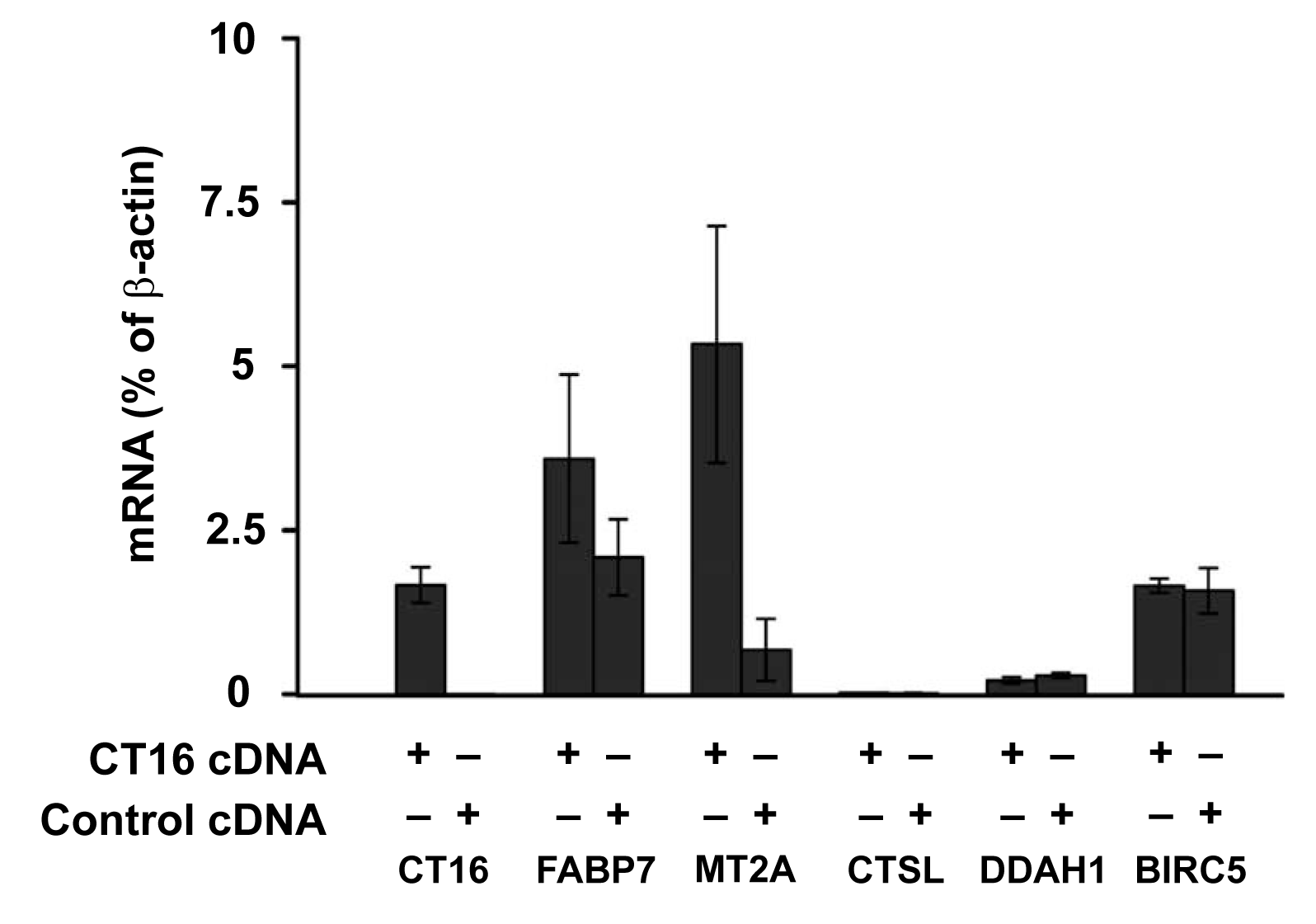

Supplement: Figure S4 — Verification of the effect CT16 overexpression on selected genes. The effect of CT16 overexpression on the mRNA levels of FABP7 and MT2A, which had the rank product FDR value<0.01 could be verified by qRT-PCR while the differential expression of CTSL, DDAH1 and BIRC5 (FDR>0.01) was not repeatable. (TIF) [file pone.0045382.s004.tif]

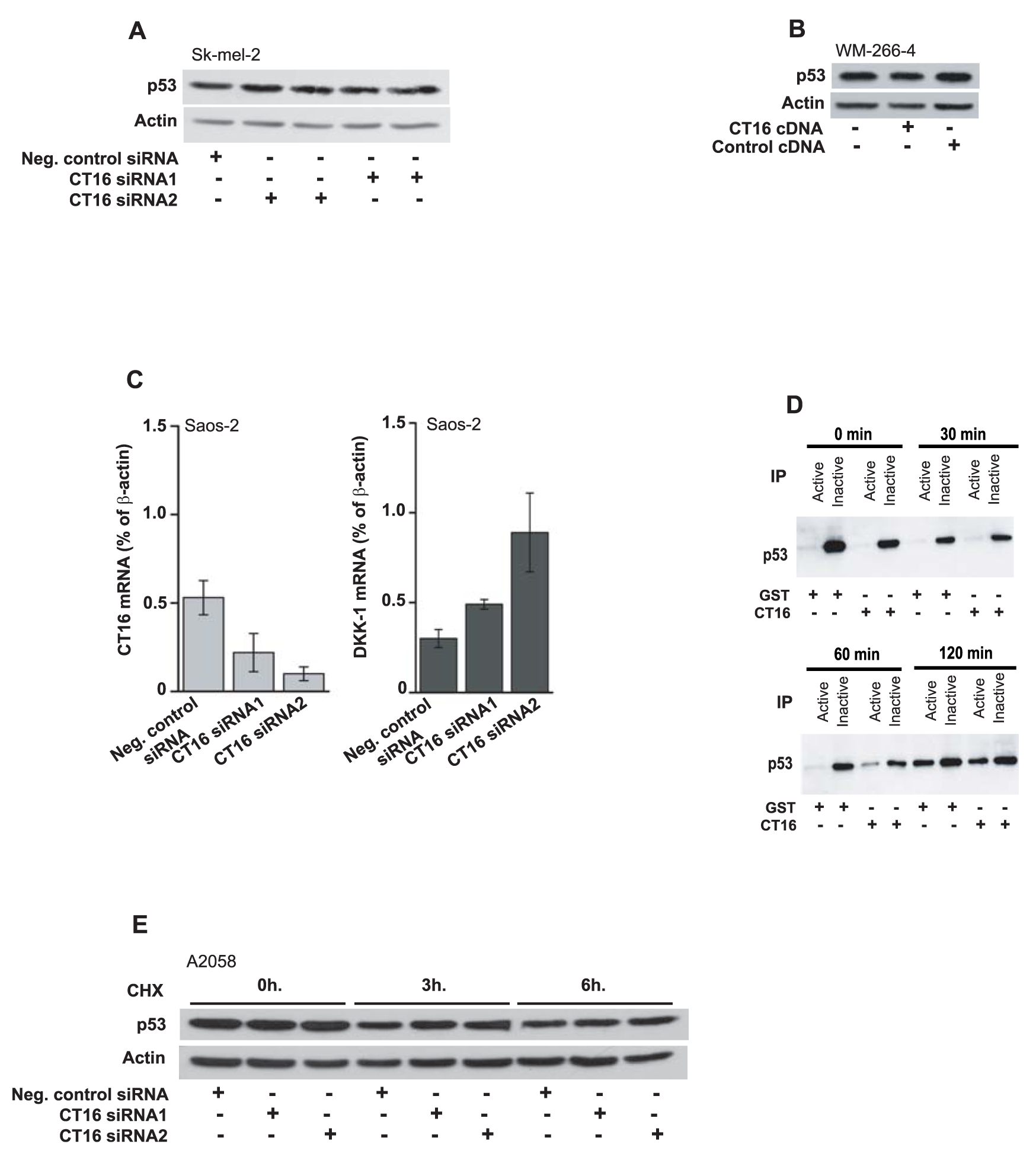

Supplement: Figure S5 — Testing the effect of CT16 on p53. (A) Sk-mel-2 cells were treated with CT16 siRNAs for 48 hours and immunoblotted for total p53 expression. (B) Western blot analysis of total p53 expression in stably transfected WM-266-4 cells, expressing CT16, and control cDNA cells, expressing empty vector. (C) DKK-1 mRNA expression was analyzed with qRT-PCR in CT16-siRNA (48 hours) transfected Saos-2 cells. Saos-2 are p53- negative cells. (D) The cell lysate from WM-266-4 cells was incubated at 37°C in the presence of either CT16 or G8T (50 µg/ml) for indicated periods of time. After incubation, the active and inactive p53 were immunoprecipitated with conformation specific antibodies, and the immunoprecipitates were analyzed by immunoblotting with p53 specific antibody. (E) A2058 cells were treated with CT16 siRNAs for 48 hours, followed by addition of 30 µg/ml CHX. After addition, cells were lysed at indicated timepoints and immunoblotted. (TIF) [file pone.0045382.s005.tif]

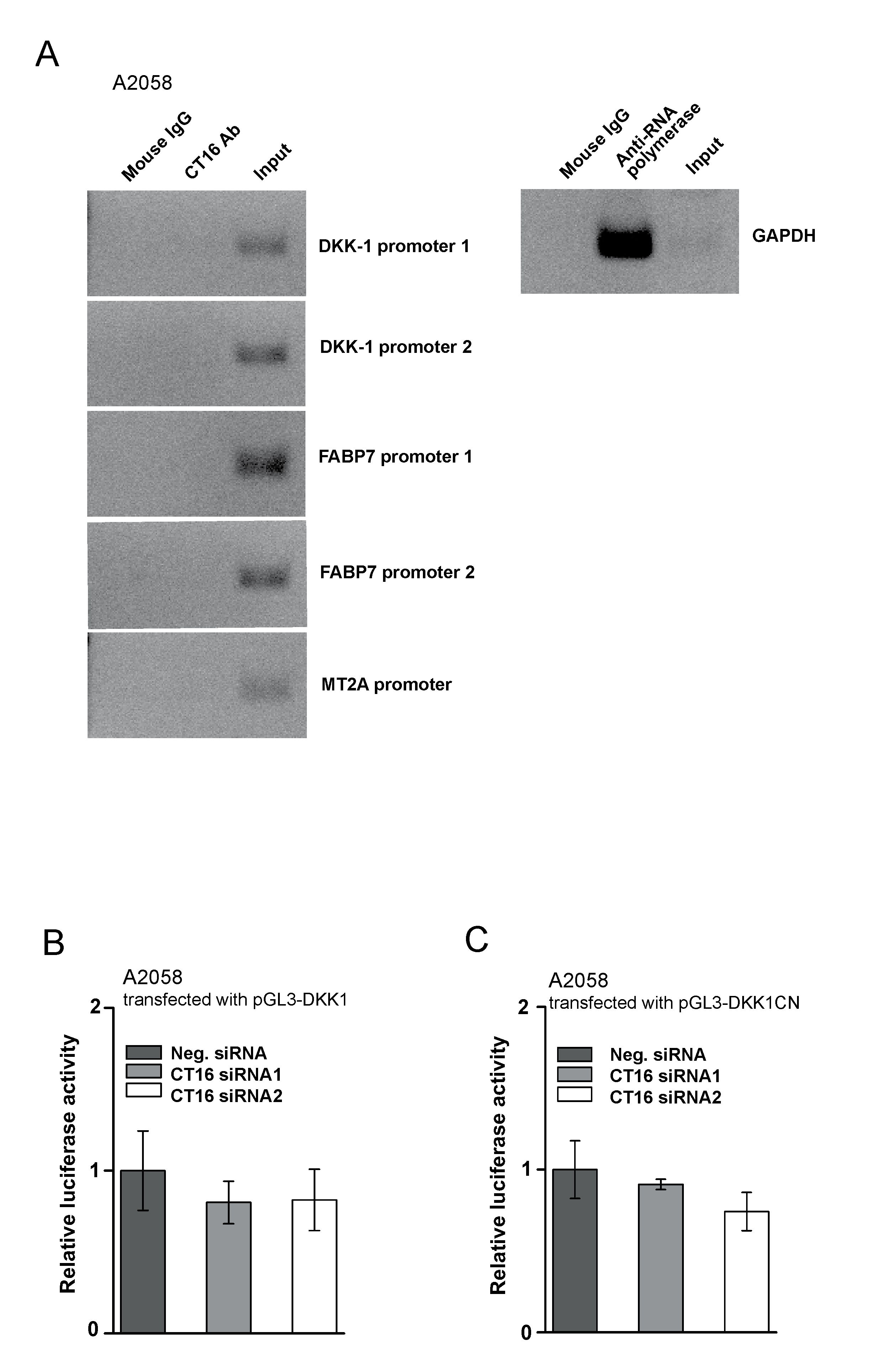

Supplement: Figure S6 — The influence between CT16 and promoters of CT16-regulated genes. (A) No interaction between CT16 and the indicated promoters of CT16-regulated genes in A2058 cells detected by chromatin immunoprecipitation. Anti-RNA Polymerase was served as a positive control. (B, C) A2058 cells transfected with either pGL3-DKK1 or pGL3-DKK1CN, gave no significant change in the relative luciferase activity between neg. siRNA and the CT16 siRNA samples. The error bars represent standard deviations from three independent experiments. (TIF) [file pone.0045382.s006.tif]
