# Supplementary material for: Melanoma-Associated Cancer-Testis Antigen 16 (CT16) Regulates the Expression of Apoptotic and Antiapoptotic Genes and Promotes Cell Survival
Source: PLoS One. 2012 Sep 21;7(9):e45382. doi: 10.1371/journal.pone.0045382 (PMC3448647; doi:10.1371/journal.pone.0045382)
Supplement: Table S1 — Comparison of chromatin immunoprecipitation data with differentially regulated genes in transfected WM-266-4 cells. (PDF) [file pone.0045382.s007.pdf]

Table S1. Comparison of chromatin immunoprecipitation data with differentially regulated genes in transfected WM-266-4 cells

Experiment	cDNA	Sample-specific genes	Common genes with CT16 upregulated genes	Common genes with CT16 downregulated genes
Histone acetylation	CT16	202	6	1
Histone acetylation	Control	329	0	5
DNA methylation	CT16	778	6	6
DNA methylation	Control	946	2	6
