# Supplementary material for: Melanoma-Associated Cancer-Testis Antigen 16 (CT16) Regulates the Expression of Apoptotic and Antiapoptotic Genes and Promotes Cell Survival
Source: PLoS One. 2012 Sep 21;7(9):e45382. doi: 10.1371/journal.pone.0045382 (PMC3448647; doi:10.1371/journal.pone.0045382)
Supplement: Table S2 — Primers and probes designed for qRT-PCR. (PDF) [file pone.0045382.s008.pdf]

Table S2. Primers and probes designed for qRT-PCR

Gene	Forward primer	Reverse primer	Probe
β -actin	5´-CGAGCGCGGCTACAGCTT	5´-TCCTTAATGTCACGCACGATTT	5´-ACGACCACGGCCGAG CGG
DKK1	5´-AAGATCACCATCAAGCCAGTAATTC	5´-AAAAGGAGTTCACTGCATTTGGAT	5´-AGGCTTCACACTTGTCAGA GACACTAAACCAGC
CTSL	5´-CAGCAAGGATGAGTGTAGGATTCA	5´-GGCGCGTGACTGGTTGAG	5´-ACCTGTGTCCACCCG
